# Coronary vessels and cardiac myocytes of middle-aged rats demonstrate regional sex-specific adaptation in response to postmyocardial infarction remodeling

**DOI:** 10.1186/2042-6410-5-1

**Published:** 2014-01-03

**Authors:** Eduard I Dedkov, Kunal Oak, Lance P Christensen, Robert J Tomanek

**Affiliations:** 1 Department of Biomedical Sciences, New York Institute of Technology College of Osteopathic Medicine, Rockefeller Building, Room 215E, Northern Boulevard, Old Westbury, NY 11568-8000, USA; 2 Department of Anatomy and Cell Biology, University of Iowa Carver College of Medicine, Iowa City, IA 52242, USA

**Keywords:** Sex-related differences, Myocardial infarction, Left ventricular remodeling, Myocardial fibrosis, Cardiac myocyte hypertrophy, Coronary angiogenesis, Coronary arteriogenesis

## Abstract

**Background:**

An increasing body of evidence indicates that left ventricular (LV) remodeling, especially the degree of reactive myocardial hypertrophy after myocardial infarction (MI), differs in males and females. Surprisingly, to date, the sex-specific post-MI alterations of the coronary vasculature remain undetermined. Therefore, we tested the hypothesis that adaptive coronary arteriolar and capillary modifications occurring in response to reactive myocyte hypertrophy differ between middle-aged male and female post-MI rats.

**Methods:**

A large MI was induced in 12-month-old male (M-MI) and female (F-MI) Sprague–Dawley rats by ligation of the left coronary artery. Four weeks after surgery, rats with transmural infarctions, greater than 50% of the LV free wall (FW), were evaluated. Sham-operated male (M-Sham) and female (F-Sham) rats served as an age-matched controls.

**Results:**

F-MI and M-MI rats had similar sized infarcts (61.3% ± 3.9% vs. 61.5% ± 1.2%) and scale of LV remodeling, as indicated analogous remodeling indices (1.41 ± 0.11 vs. 1.39 ± 0.09). The degree of reactive post-MI myocardial hypertrophy was adequate to normalize LV weight-to-body weight ratio in both sexes; however, the F-MI rats, in contrast to males, showed no myocyte enlargement in the LVFW epimyocardium. At the same time, a greater than 50% expansion of myocyte area in the male epimyocardium and in the female endomyocardium was accompanied by a 23% (*P* < 0.05) increase in capillary-to-myocyte ratio, indicative of adaptive angiogenesis. Based on arteriolar length density in post-MI hearts, the resistance vessels grew in the male LVFW as well as the septum by 24% and 29%, respectively. In contrast, in females, a significant (30%) expansion of arteriolar bed was limited only to the LVFW. Moreover, in F-MI rats, the enlargement of the arteriolar bed occurred predominantly in the vessels with diameters <30 μm, whereas in M-MI rats, a substantial (two- to threefold) increase in the density of larger arterioles (30 to 50 μm in diameter) was also documented.

**Conclusion:**

Our data reveal that while both sexes have a relatively similar pattern of global LV remodeling and adaptive angiogenesis in response to a large MI, male and female middle-aged rats differ markedly in the regional scale of reactive cardiac myocyte hypertrophy and adaptive arteriogenesis.

## Background

A large transmural myocardial infarction (MI) of the left ventricle (LV) initiates progressive structural remodeling, consisting of scarring and thinning of the infarcted region, dilatation of the LV chamber, reactive fibrosis, and compensatory hypertrophy in the noninfarcted LV myocardium [1-4]. It has also been established that the compensatory growth of cardiac myocytes in noninfarcted LV myocardium is often accompanied by the adaptive growth of coronary microvessels (capillaries and/or arterioles) [5,6]. The anatomical expansion of the vascular bed occurs primarily in the areas containing enlarged cardiac myocytes and compromised blood perfusion [7-9], indicating a strong functional link between the post-MI myocardial growth and the adaptive expansion of coronary vasculature [10].

In recent years, the accumulating body of knowledge derived from experimental animal studies and clinical research has revealed that the adaptive responses to post-MI remodeling differ between males and females [11]. For example, female rats and mice with a large transmural MI had less compensatory hypertrophy in noninfarcted LV regions than did males [12-14]. In concert with these experimental data, studies on humans have also demonstrated that women with LV dysfunction, caused in part by previous MI, were characterized by a smaller increase in LV mass compared to their respective male counterparts [15,16]. Surprisingly, despite the fact that males and females differ markedly in the extent of post-MI compensatory myocardial growth, there are no published studies investigating the degree of the adaptive coronary vessel growth between the sexes.

Furthermore, since MI is most common in middle-aged and senescent individuals of both genders [17,18], the analysis of adaptive changes in post-MI hearts of middle-aged, rather than young, animals is of greater relevance to the human population. Unfortunately, the majority of previously conducted MI experiments utilized either young or young adult animals. However, aged experimental animals, such as mice and rats, have been shown to differ markedly from their younger counterparts with regard to post-MI remodeling, including the level of LV hypertrophy [19,20], the degree of reactive myocardial fibrosis [20], and adaptive coronary vessel growth [6,21,22].

Accordingly, the aim of the current study was to test the hypothesis that sex is a determinant of adaptive responses of cardiac myocytes and coronary microvessels (capillaries and arterioles) in the noninfarcted LV myocardium after a large transmural MI in middle-aged rats.

## Methods

All animal procedures were performed in accordance with the *Guide for the Care and Use of Laboratory Animals* published by the US National Institute of Health (NIH Publications No. 85–23, revised 1996) and approved by the University of Iowa Animal Care and Use Committee.

### Animals and experimental model

All experiments were conducted on 12-month-old (middle-aged) Sprague–Dawley rats of both sexes (Harlan, Indianapolis, IN, USA). Although in this study we did not assess plasma estradiol levels in our female animals, previous studies have determined that at this age, the rats are usually premenopausal but start to exhibit irregular estrous cycles [23,24] similar to middle-aged women during the perimenopausal period [25].

Female (F-MI, *n* = 9) and male (M-MI, *n* = 9) rats were anesthetized with a mixture of ketamine (100 mg/kg i.p.) and xylazine (10 mg/kg i.p.) and the left coronary artery was permanently ligated near its origin, as previously detailed [26]. The mortality rate among post-MI rats was approximately 22% in males and approximately 11% in females, with all deaths occurring within the first 48 h after surgery. Sham-operated female (F-Sham, *n* = 6) and male (M-Sham, *n* = 7) rats served as age-matched controls. Following surgery, the rats were housed under climate-controlled conditions at a 12-h light/dark cycle and provided with standard rat chow and water *ad libitum*.

**Figure 1:**
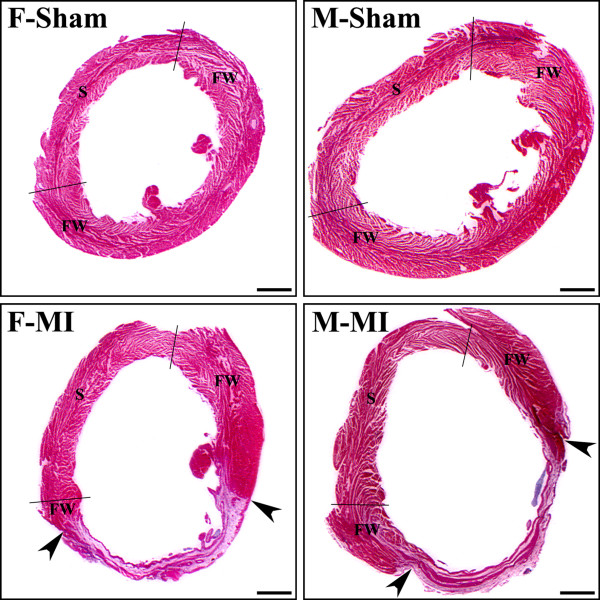
**H&E-stained cross sections of the left ventricle from sham and MI hearts of the rats.** Female (F) and male (M). In each image, two black lines separate the interventricular septum (S) from the LV free wall (FW). In post-MI hearts, the arrowheads indicate the region of a transmural scar. Scale bars are 1.5 mm.

The rats were sacrificed 4 weeks after surgery, and the hearts were processed for comprehensive morphological analysis. All data from the noninfarcted LV free wall (FW) were derived from tissue approximately 1.5 to 2 mm distal from the infarct edge (Figure 1).

### Ventricular weight measurement, infarct size estimation, and tissue sampling

The heart was arrested in diastole by the infusion of 2% lidocaine HCl into the left ventricle, then excised and perfuse-fixed on a Langendorff apparatus under constant pressure (100 mmHg) for 20 min with 4% paraformaldehyde (PFA) in phosphate-buffered saline (PBS). The hearts were stored in a fresh 4% PFA solution for 24 h at +4°C. The atria were removed, and the right ventricular FW and the left ventricle (LVFW plus septum) were briefly blotted dry with filter paper and separately weighed. The left ventricle was then cut transversely into five parallel slices with a multiblade guillotine.

In hearts with MI, the all LV slices were digitized and infarct size was estimated using Image-Pro Analyzer 7.0 software (Media Cybernetics, L.P., Silver Spring, MD, USA), as detailed previously [26]. Briefly, in each digitized slice, the lengths of the entire free wall and its portion occupied by the scar (both obtained at the midwall level) were measured, and the extent of the scarred area was estimated as the ratio between scar length and length of the entire free wall. The mean of these ratios was calculated for each heart. Finally, the infarct size was expressed as a percentage of the LVFW. The data from post-MI rats of both sexes were included in the study only if the infarct size was equal or greater than 50% of the LVFW. According to infarct size measurements, the seven hearts from M-MI rats and six hearts from F-MI rats were selected for final evaluation.

From each heart, two midventricular slices (at the level of the papillary muscles) were processed and embedded in paraffin, because they are most representative of the scale of LV remodeling and the extent of the transmural scar [27].

### Histology, immunohistochemistry, and microscopy

Transverse 8.0-μm-thick serial sections were cut from paraffin-embedded LV slices and mounted onto microscope slides. Hematoxylin and eosin (H&E) and Masson's trichrome stains were used for general morphological evaluation. Picrosirius red stain was utilized to visualize interstitial and periarteriolar collagen fibrils.

Additional sections were either immunostained with a Cy3-conjugated anti-smooth muscle (SM) α-actin antibody (Sigma, St. Louis, MO, USA) to visualize the arterioles or double-labeled with a mixture of an Alexa Fluor 594-conjugated Bandeiraea Simplicifolia-I (BSI) isolectin B4 (Molecular Probes, Inc., Eugene, OR, USA) and anti-laminin antibody (Sigma, St. Louis, MO, USA) to visualize capillaries and to outline the cardiac myocytes, as detailed previously [6,21,26]. An Alexa Fluor 488-conjugated goat anti-rabbit antibody (Molecular Probes, Inc., Eugene, OR, USA) was used for visualization of a primary anti-laminin antibody. All sections were counterstained with DAPI (4′, 6-Diamidino-2-Phenylindole; Vector Laboratories, Inc., Burlingame, CA, USA) to visualize the cell nuclei.

The stained sections were examined under the Olympus BX53 microscope (Shinjuku, Tokyo, Japan), and light and fluorescence images were captured into a computer using an Olympus DP72 digital camera (Shinjuku, Tokyo, Japan).

### Morphometric and stereological analyses

Morphometric and stereological analyses were conducted on digitized images using Image-Pro Analyzer 7.0 software (Media Cybernetics, Inc., Bethesda, MD, USA).

The H&E and Masson's trichrome-stained sections were used to obtain the following parameters of the left ventricle: (1) LV cross-sectional area (CSA) and diameter, (2) LV cavity CSA and mean cavity diameter, and (3) mean thickness of the LVFW and septum. From these measurements, we calculated the LV cavity diameter to septum thickness ratio [14] and the remodeling index detailed previously by Boyle and Weismann [28], i.e., (LV cavity CSA of MI heart/Average LV cavity CSA of Sham hearts) × (LVFW thickness of MI heart/Average LVFW thickness of sham hearts).

The picrosirius red-stained sections were used to identify fibrillar collagen in interstitial and periarteriolar spaces. The interstitial collagen content was estimated as the volume fraction of the area occupied by fibrillar collagen and cardiac myocytes. The periarteriolar collagen content was defined as the ratio between the area occupied by the fibrillar collagen within the area surrounding the resistance vessel and the vessel area itself (media + lumen), as detailed previously [21,22]. For each heart, 14 to 20 resistance vessels (5 to 55 μm in diameter) per myocardial region (i.e., LVFW and septum) were randomly selected and evaluated.

SM α-actin-positive vessels (10 to 50 μm in diameter) were used to calculate the arteriolar length, volume, and numerical densities, whereas vessels labeled with BSI-B4 isolectin (less than 5 μm in minimal diameter) were used to estimate capillary numerical density, as previously detailed [21,26]. Arteriolar tortuosity (or an anisotropy coefficient) was then calculated as follows: arteriolar length density divided by arteriolar numerical density [29]. Cross-sectional area and numerical density of laminin-outlined transversely cut cardiac myocytes were determined in the same regions used for capillary analysis, and a capillary-to-myocyte ratio was calculated based on the numerical densities calculated for capillaries and cardiac myocytes, as previously detailed [21,26]. All parameters were estimated separately for the following three myocardial regions: LV epi- and endomyocardium and the septal endomyocardium. Only the areas in which the cardiac myocyte profiles demonstrated the relatively transverse plane and the presence of centrally located nuclei were used for evaluation. On average, approximately 280 to 310 myocyte profiles per region were counted in each heart.

### Statistical analysis

Data are expressed as the mean ± SEM. Statistical analysis was performed using IBM SPSS Statistics 22.0 software package (IBM Corp., Armonk NY, USA). Two-way analysis of variance (ANOVA) was performed to determine the interaction effect of sex (males vs. females) and the experimental model (Sham vs. MI) on the dependent variables. If a statistically significant difference was detected in the interaction effect and/or the main effects of sex and experimental model, a *post hoc* analysis was performed using Student's *t* test with Bonferroni multiple comparison correction. *P* < 0.05 was selected to denote significant differences.

## Results

Considering the fact that middle-aged female and male rats had significantly different body weights, left and right ventricular weights as well as LV chamber dimensions (Table 1), our main emphasis was to conduct side-by-side comparison of sex-specific adaptations that occurred in the left ventricle of each sex group during cardiac remodeling triggered by a large transmural MI.

**Table 1:** Global parameters of the left ventricle in shame and MI hearts from male (M) and female (F) rats

	**M-Sham**	**M-MI**	**F-Sham**	**F-MI**
Number	7	7	6	6
Infarct size, % of LVFW	-	61.5 ± 1.2 (57.5–63.8)	-	61.3 ± 3.9 (50.0–71.3)
BW, g	560.0 ± 15.4	536.8 ± 27.2	362.5 ± 6.4^§§§^	335.7 ± 21.5^†††^
VW, mg	1476.4 ± 39.7	1461.8 ± 88.2	1214.5 ± 50.6^§^	1084.2 ± 16.6^†††^
LVW, mg	1150.4 ± 33.4	1100.4 ± 55.2	935.2 ± 46.0^§§^	829.5 ± 12.2^†††^
RVW, mg	326.0 ± 9.6	361.4 ± 40.5	279.3 ± 5.6	254.7 ± 18.3^†^
LVW/BW, mg/g	2.06 ± 0.08	2.05 ± 0.07	2.58 ± 0.09^§^	2.52 ± 0.15^†^
LVW/VW, mg/mg	0.78 ± 0.01	0.76 ± 0.02	0.77 ± 0.01	0.77 ± 0.01
LV diameter, mm	11.06 ± 0.09	11.03 ± 0.25	9.89 ± 0.24^§^	9.88 ± 0.19^††^
LV CSA, mm^2^	96.9 ± 1.6	96.5 ± 4.3	78.3 ± 4.0^§^	78.4 ± 3.1^†^
LV cavity diameter, mm	6.33 ± 0.30	7.58 ± 0.22^*^	5.82 ± 0.34	6.61 ± 0.44^†^
LV cavity CSA, mm^2^	32.9 ± 2.9	46.4 ± 2.7^*^	27.9 ± 3.1	36.4 ± 4.5^†^
LVFW thickness, mm	2.31 ± 0.13	2.29 ± 0.11	1.92 ± 0.10	2.12 ± 0.21
Septum thickness, mm	1.75 ± 0.09	1.77 ± 0.12	1.64 ± 0.08	1.68 ± 0.11
LV cavity diameter/septum	3.67 ± 0.30	4.39 ± 0.35	3.60 ± 0.30	4.02 ± 0.44
Remodeling index	-	1.39 ± 0.09	-	1.41 ± 0.11

Values are the mean ± SEM; *n*, Number of rats; LVFW, left ventricular free wall; BW, body weight; VW, ventricular weight; LVW, left ventricular weight; RVW, right ventricular weight; CSA, cross-sectional area. Remodeling index = (LV cavity CSA of MI heart/Average LV cavity CSA of sham hearts) × (LVFW thickness of MI heart/Average LVFW thickness of sham hearts). ^*^P < 0.05 vs. corresponding Sham group; ^§^*P* < 0.05, ^§§^*P* < 0.01, and ^§§§^*P* < 0.001 vs. M-Sham; ^†^*P* < 0.05, ^††^*P* < 0.01, and ^†††^*P* < 0.001 vs. M-MI.

### LV structural remodeling and myocardial fibrosis

Four weeks after left coronary artery occlusion, the mean infarct size was comparable between male and female rats (Table 1). The loss of cardiac myocytes was compensated by LV hypertrophy in both post-MI groups, as indicated unchanged LV weight-to-body weight ratios, that were similar to a corresponding group of sham rats. Furthermore, unaltered LV weight-to-ventricular weight ratios revealed that the left ventricles of both sexes had undergone a relatively proportional enlargement during 4 weeks of post-MI remodeling. It also appears that the compensatory LV growth in male and female rats was primarily longitudinal, since the transverse LV chamber dimensions and thickness of the noninfarcted myocardial wall remained relatively similar to the sham values. Most importantly, even though male post-MI rats, in contrast to female animals, showed a significant expansion of the LV cavity (Figure 1), a global pattern of LV chamber remodeling remained comparable between two sexes, as indicated by nearly identical values for remodeling index and LV cavity diameter to septum thickness ratio (Table 1).

**Figure 2:**
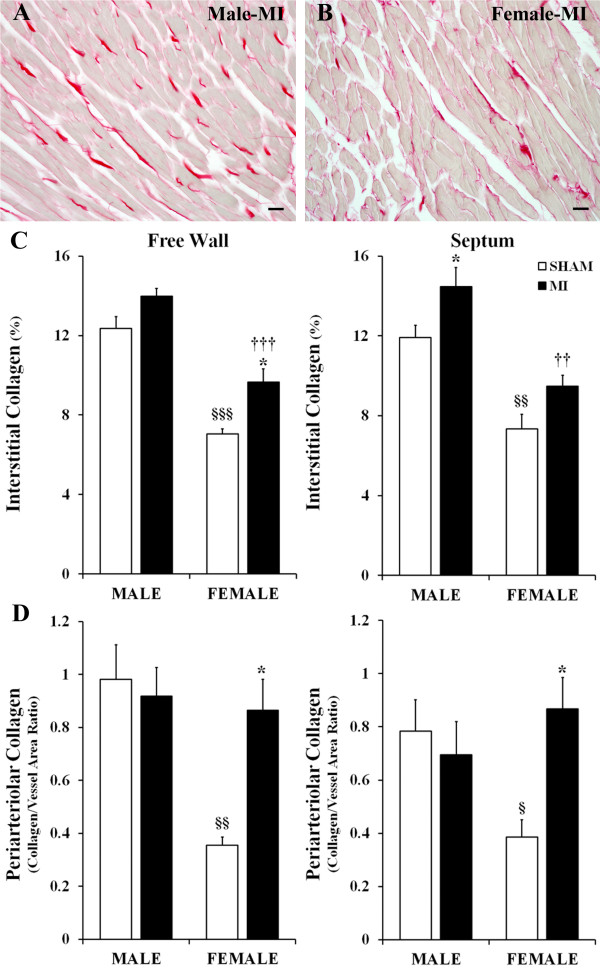
**Extent of interstitial and periarteriolar fibrosis. (A,B)** Representative images of picrosirius red-stained collagen fibers (bright red color) in the LV free wall myocardium of post-MI male and female rats. **(C)** Fractional volume of interstitial collagen fibers in the LV free wall and septum. **(D)** The content of periarteriolar collagen in the LV free wall and septum. Scale bars are 20 μm. Values are the mean ± SEM; *n* = 7 male rats/group; *n* = 6 female rats/group. A two-way ANOVA revealed a statistically significant interaction between the effects of sex and the experimental model on perivascular collagen content in both LV free wall, *F* (1, 22) = 6.860, *P =* 0.020, and the septum, *F* (1, 22) = 5.876, *P =* 0.028. ^*^P < 0.05 vs. a corresponding sham group; ^§^*P* < 0.05, ^§§^*P* < 0.01, and ^§§§^*P* < 0.001 vs. M-Sham; ^††^*P* < 0.01 and ^†††^*P* < 0.001 vs. M-MI rats.

Post-MI remodeling in the noninfarcted LV myocardium of both sexes was associated with an increase in the content of interstitial fibrillar collagen (Figure 2A,B). As shown in Figure 2C, the fractional volume of interstitial collagen was higher in post-MI rats by 13% in male and 37% in female free wall and 23% and 34% in male and female septum, respectively, as compared with corresponding sham rats (Figure 2C). In contrast, periarteriolar collagen revealed a marked (2.5- to threefold) accumulation only in post-MI female rats (Figure 2D). In spite of a more exaggerated accumulation of interstitial and periarteriolar collagen in surviving myocardium, the post-MI female rats had significantly smaller fractional volume of fibrillar collagen in both the LV free wall and septum compared to their age-matched male counterparts. This was in part due to the fact that the fractional volume of interstitial and periarteriolar collagen was much lower in female rats prior to infarction, as indicated by the values in shams (Figure 2C,D).

### Capillary bed adaptation in response to regional cardiac myocyte hypertrophy

**Figure 3:**
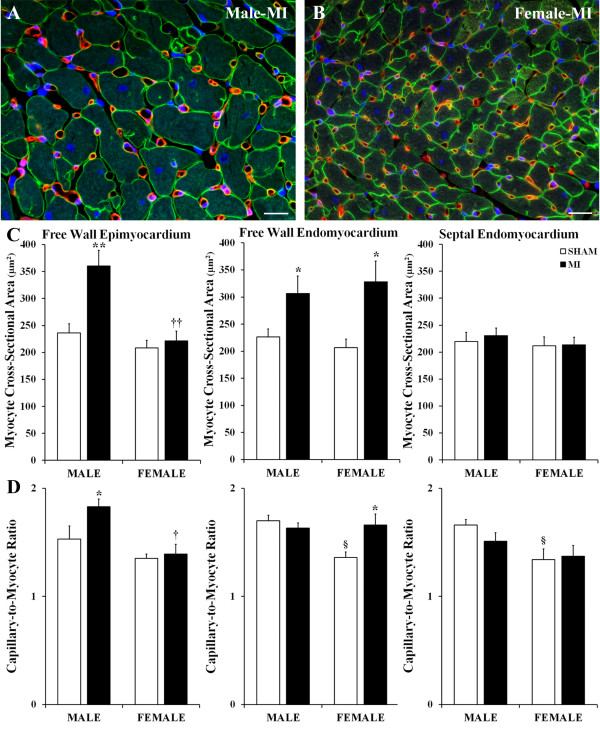
**Degree of reactive cardiac myocyte hypertrophy and compensatory angiogenesis. (A,B)** Representative images of the free wall epimyocardium of post-MI male and female hearts immunofluorescence-stained with an antibody against laminin (green color), BSI isolectin B4 (red color), and DAPI (blue). **(C)** Cross-sectional area of cardiac myocytes in the LV free wall and septum. **(D)** Capillary-to-myocyte ratios in the LV free wall and septum. Scale bars are 20 μm. Values are the mean ± SEM; *n* = 7 male rats/group; *n* = 6 female rats/group. Note, in septal myocardium, all measurements were done on the endomyocardial side that is faced toward the LV cavity. A two-way ANOVA revealed a statistically significant interaction between the effects of sex and the experimental model on myocyte CSA in epimyocardial region, *F* (1, 22) = 6.032, *P =* 0.026, and on the capillary-to-cardiac myocyte ratio in free wall endomyocardium, *F* (1, 22) = 6.307, *P =* 0.025. ^*^P < 0.05 and ^**^P < 0.01 vs. a corresponding sham group; ^§^*P* < 0.05 vs. M-Sham; ^†^*P* < 0.05 vs. M-MI rats.

Despite the fact that in both sexes, average thickness of the noninfarcted LV wall remained comparable to age-matched sham rats (Table 1), cardiac myocytes in the noninfarcted free wall myocardium of the male rats were significantly enlarged in both the epimyocardium (57%) and endomyocardium (38%) compared to shams, whereas in post-MI female rats, a marked (62%) compensatory growth of cardiac myocytes occurred only in the free wall endomyocardium (Figure 3A,B,C). While these data suggest that during the four post-MI weeks, both male and female rats experienced a substantial reduction in the number of cardiac myocytes across the LV wall, the loss of cardiac myocytes was less prominent in post-MI female rats, primarily in the free wall epimyocardium, compared to that in post-MI males (Table 2 and Figure 3A,B). At the same time, neither of the two post-MI groups revealed any concentric enlargement of cardiac myocytes in the septum (Table 2).

**Table 2:** Capillary and cardiac myocyte numerical densities and capillary-to-myocyte ratio in the left ventricle of shame and MI hearts from male (M) and female (F) rats

	**Number**	**LV free wall**			**Septum**		
		**CD, number/mm**^**2**^	**MD, number/mm**^**2**^	**C/M**	**CD, number/mm**^**2**^	**MD, number/mm**^**2**^	**C/M**
Epimyocardium							
M-Sham	7	4,929.7 ± 407.7	3,142.9 ± 193.6	1.59 ± 0.12	N/A	N/A	N/A
M-MI	7	3,818.1 ± 169.9^*^	2,178.9 ± 207.6^*^	1.81 ± 0.08^*^	N/A	N/A	N/A
F-Sham	6	5,405.5 ± 418.9	4,027.2 ± 262.2^§^	1.35 ± 0.04	N/A	N/A	N/A
F-MI	6	5,142.7 ± 773.8	3,661.4 ± 312.3^††^	1.39 ± 0.09^†^	N/A	N/A	N/A
Endomyocardium							
M-Sham	7	6,220.4 ± 329.9	3,739.6 ± 195.2	1.67 ± 0.05	6,107.4 ± 459.6	3,783.9 ± 286.5	1.62 ± 0.04
M-MI	7	4,353.3 ± 687.1^*^	2,704.7 ± 351.0^*^	1.60 ± 0.06	5,215.2 ± 592.3	3,443.6 ± 262.4	1.52 ± 0.09
F-Sham	6	5,477.4 ± 396.5	4,032.1 ± 305.2	1.36 ± 0.05^§^	5,361.5 ± 908.9	3,938.7 ± 346.8	1.34 ± 0.12^§^
F-MI	6	4,492.8 ± 648.0	2,709.2 ± 337.3^*^	1.66 ± 0.13^*^	5,510.2 ± 717.4	3,875.4 ± 359.8	1.37 ± 0.11

Values are the mean ± SEM. *n*, number of rats; CD, capillary density; MD, myocyte density; C/M, capillary-to-myocyte ratio; N/A, mot applicable. Note that in septum all parameters were analyzed only in LV endomyocardium. A two-way ANOVA revealed a statistically significant interaction between the effects of sex and the experimental model on the capillary-to-cardiac myocyte ratio in free wall endomyocardium, *F* (1, 22) = 6.307, *P =* 0.025. ^*^P < 0.05 vs. corresponding Sham group; ^§^*P* < 0.05 vs. M-Sham; ^†^*P* < 0.05 and ^††^*P* < 0.01 vs. M-MI.

Generally, the transverse enlargement of cardiac myocytes in the LV free wall of post-MI male and female rats was associated with the reduction in capillary density (Table 2). Nevertheless, in both sexes, a greater than 50% expansion in myocyte cross-sectional area was accompanied by a significant compensatory angiogenic response. As shown in Figure 3D, the capillary-to-myocyte ratio was 23% greater in both the male epimyocardium and the female endomyocardium, as compared to corresponding sham rats. At the same time, the unchanged values of capillary-to-myocyte ratio detected in other areas of the remodeled LV myocardium of both sexes (Table 2) suggested an adequate functional match between the cardiac myocyte size and the capillary density in these regions.

### Regional differences in arteriolar bed adaptation

**Figure 4:**
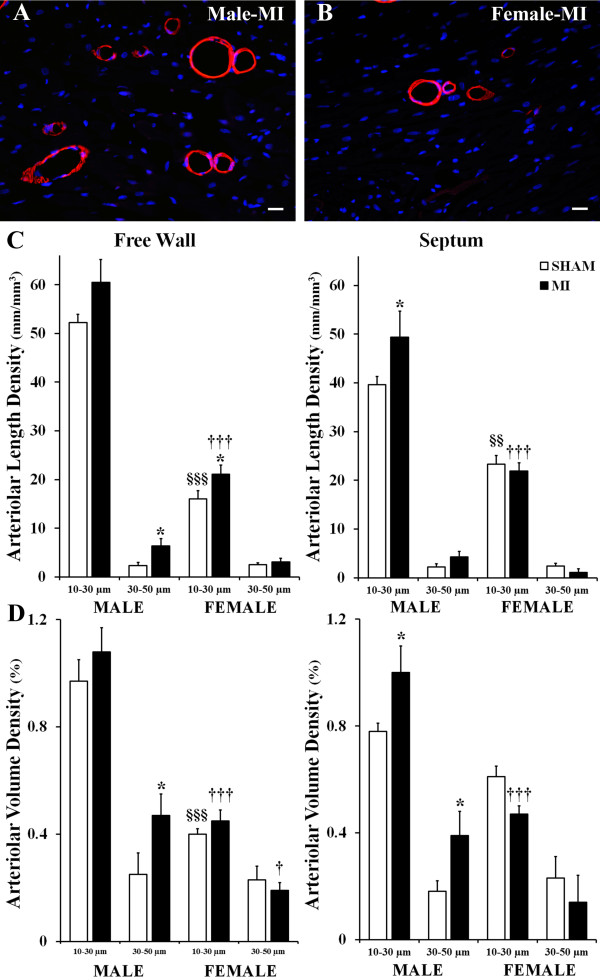
**Scale of adaptive arteriogenesis. (A,B)** Representative images of the LV free wall myocardium of post-MI male and female rats immunofluorescence-stained with an antibody against smooth muscle α-actin (red color) and DAPI (blue). **(C)** Arteriolar length density relative to a vessel diameter group in the LV free wall and septum. **(D)** Arteriolar volume density relative to a vessel diameter group in the LV free wall and septum. Scale bars are 20 μm. Values are the mean ± SEM; *n* = 7 male rats/group; *n* = 6 female rats/group. ^*^P < 0.05 vs. a corresponding sham group; ^§§^*P* < 0.01 and ^§§§^*P* < 0.001 vs. M-Sham rats; ^†^*P* < 0.05 and ^†††^*P* < 0.001 vs. M-MI rats.

Post-MI remodeling of noninfarcted LV myocardium was associated with a substantial expansion of the coronary arteriolar bed in both sexes as indicated by a significant increase in arteriolar length density compared to corresponding sham values (Table 3). However, while in male rats, arteriolar density increased in both the LV free wall and the septum by 24% and 29%, respectively, in females, a significant (30%) expansion of the arteriolar bed occurred only in the LV free wall (Figure 4A,B,C). Furthermore, whereas in female rats arteriolar growth occurred predominantly in the vessels with diameters <30 μm, in male rats, a substantial (two to threefold) increase occurred in larger arterioles (30 to 50 μm in diameter) as well (Figure 4D).

**Table 3:** Arteriolar diameter, numerical density, length density, volume densities, and the degree of arteriolar tortuosity in the left ventricle of shame and MI hearts from male (M) and female (F) rats

	**Number**	**Diameter minimum, μm**	***N***_**A**_**, counts/mm**^**2**^	***L***_**V**_**, mm/mm**^**3**^	**Tortuosity, *L***_**V**_**/*N***_**A**_	***V***_**V**_**, percent**

LV free wall						
M-Sham	7	15.7 ± 0.6	20.4 ± 1.3	54.6 ± 2.3	2.7 ± 0.1	1.22 ± 0.16
M-MI	7	16.4 ± 0.4	22.2 ± 1.4	66.8 ± 4.8^*^	3.0 ± 0.1	1.55 ± 0.15
F-Sham	6	19.5 ± 0.7^§§§^	6.9 ± 0.3^§§§^	18.6 ± 1.6^§§§^	2.7 ± 0.2	0.62 ± 0.03^§^
F-MI	6	17.0 ± 0.4^*^	9.3 ± 0.8^†††^	23.7 ± 2.2^*†††^	2.6 ± 0.2	0.63 ± 0.06^††^
Septum						
M-Sham	7	15.7 ± 0.3	17.5 ± 1.4	41.9 ± 1.6	2.4 ± 0.1	0.96 ± 0.05
M-MI	7	16.2 ± 0.3	21.2 ± 1.9	53.6 ± 5.7^*^	2.5 ± 0.1	1.38 ± 0.11^*^
F-Sham	6	17.9 ± 0.5^§§^	11.0 ± 1.1^§^	25.8 ± 1.4^§§^	2.4 ± 0.2	0.84 ± 0.09
F-MI	6	16.8 ± 0.6	8.9 ± 0.9^†††^	23.0 ± 2.2^†††^	2.6 ± 0.1	0.61 ± 0.12^†††^

Values are the mean ± SEM. *n*, number of rats; *N*_A_, numerical density; *L*_V_, length density; *V*_V_, volume density. A two-way ANOVA revealed a statistically significant interaction between the effects of sex and the experimental model on the arteriolar diameter in LV free wall, *F* (1, 22) = 8.943, *P =* 0.009, and on arteriolar volume density in septum, *F* (1, 22) = 10.903, *P =* 0.005. ^*^P < 0.05 vs. corresponding Sham group; ^§^*P* < 0.05, ^§§^*P* < 0.01, and ^§§§^*P* < 0.001 vs. M-Sham; ^†^*P* < 0.05, ^††^*P* < 0.01, and ^†††^*P* < 0.001 vs. M-MI.

**Figure 5:**
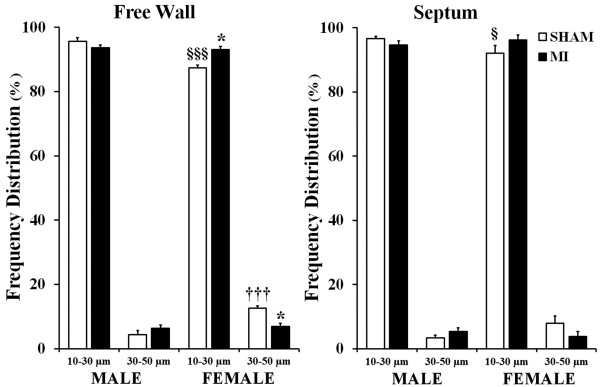
**Frequency distribution of arterioles relative to a vessel diameter group.** Values are the mean ± SEM; *n* = 7 male rats/group; *n* = 6 female rats/group. A two-way ANOVA revealed a statistically significant interaction between the effects of sex and the experimental model on the frequency distribution of arterioles in LV free wall, *F* (1, 22) = 12.001, *P =* 0.003. ^*^P < 0.05 vs. a corresponding sham group; ^§^*P* < 0.05 and ^§§§^*P* < 0.001 vs. M-Sham rats; ^†††^*P* < 0.001 vs. M-MI rats.

It is important to emphasize the fact that although the arteriolar beds of the female sham and post-MI rats were markedly smaller than in the hearts of their age-matched male counterparts, we found a relatively similar pattern of arteriolar network organization in two sexes, as indicated by a comparable degree of arteriolar tortuosity (Table 3) and the nearly analogous scale of arteriolar frequency distribution in relation to a particular vessel diameter group (Figure 5). However, in contrast to male rats, post-MI females showed a more significant shift in hierarchic distribution of arterioles within the vascular bed, especially in the LV free wall, as compared to sham rats. Such sex-specific arteriolar tree modification was further exposed by the opposing patterns of changes within the large and small diameter vessel groups in two sexes (Figure 5).

## Discussion

Data from this study provide four major findings. First, male and female middle-aged rats with a transmural MI of the comparably large size undergo a relatively similar pattern of global LV chamber remodeling and reactive myocardial hypertrophy; however, the myocardial content of fibrillar collagen, especially in periarteriolar regions, increases more in female than in male rats. Second, the transverse growth of cardiac myocytes in the LV free wall is limited to the endomyocardium of female post-MI rats, whereas male rats experience a marked enlargement of cardiac myocyte in both epimyocardial and endomyocardial regions. Third, post-MI rats of both sexes showed that significant adaptive angiogenesis occurs only in the areas of the LV free wall associated with the cross-sectional expansion of cardiac myocyte greater than 50%. Finally, a significant expansion of the coronary arteriolar tree occurs in both the LV free wall and the septum of post-MI male rats, whereas in females, adaptive arteriogenesis is limited only to the free wall. Taken together, these findings support our conclusion that significant regional sex-related differences characterize the post-MI adaptive responses of various structural components in surviving LV myocardium, especially cardiac myocytes and coronary microvessels.

### Does sex/gender affect the pattern of global post-MI LV remodeling and a regional hypertrophic response of the cardiac myocytes?

Although the structural alterations in LV architecture, termed ventricular remodeling, which occur in response to a large transmural MI, have been studied for the last three decades in both humans and experimental animals [1-3,30], the findings related to sex/gender-specific differences during this process remain obscure [11,31,32]. Therefore, our study is the first to conduct a side-by-side comparison of the global and regional patterns of LV remodeling between post-MI male and female middle-aged rats. We focused specifically on middle-aged animals because this age group, along with older individuals, corresponds to a human population, including both sexes, which are most vulnerable for myocardial ischemia and infarction [17,18,33,34]. We also chose to evaluate the hearts at the end of the first post-MI month because it usually represents a compensated phase of the infarct-induced LV remodeling process. At this stage, the left ventricle contains a formed scar [32], hypertrophied cardiac myocytes, and significantly expanded cavity [2], but yet preserves the thickness of the noninfarcted myocardial wall [35] and the external cross-sectional chamber dimensions [36]. In agreement with these observations, we found that although the LV cavity of post-MI male and female middle-aged rats was expanded, the LV weight-to-body weight ratio, the external cross-sectional dimensions of the LV chamber, and the thickness of both the septal and noninfarcted free wall myocardium remained similar to shame values, suggesting a compensated stage of ventricular remodeling. Most importantly, we established the fact that despite a significant difference in the size of the left ventricles, the post-MI middle-aged male and female rats had virtually identical values of the remodeling indices and the LV cavity diameter to septum thickness ratio, indicative of a similar pattern of global LV remodeling between the sexes.

However, our data are in contrast to previous observations by others who studied young adult post-MI rats [12,14,31]. These studies noted a significant difference in the LV remodeling pattern between male and female animals. One study found a greater increase in the thickness of noninfarcted free wall in males compared to females [12], whereas another reported that males had a smaller cross-sectional expansion of the LV chamber than females [31]. We believe that discrepancy between our findings and those reported previously are attributable to the significant difference in the age of rats studied. This argument is supported by findings showing that LV parameters such as cavity dimensions, the size of cardiac myocytes, and the scale of myocardial fibrosis differ significantly between young and aged rodents (mice and rats) both at the onset of MI and during LV remodeling [19,20]. These data suggest that the high level of myocardial fibrosis in the hearts of sham-operated middle-aged rats, particularly males, is probably age-related since data from younger animals did not reveal extensive fibrosis [13]. Yet, our data are consistent with the fact that LV myocardium of the female rats, even after a large MI, revealed a lesser amount of interstitial collagen than males [13]. To a certain extent, this phenomenon can be attributed to an antifibrotic effect of estrogen [37], the level of which remains fairly high in middle-aged rats [23]. However, we found a significant interaction between the effects of sex and the experimental model on perivascular collagen content in both LV free wall and the septum. Particularly, we determined that post-MI remodeling in middle-aged female rats causes a much greater accumulation of interstitial and especially perivascular collagen than in males.

Since the compensatory recovery of LV weight in post-MI male and female middle-aged rats occurred without noticeable thickening of the myocardial wall, it is likely that in both sexes, most of the compensatory myocardial growth occurred via cardiac myocyte lengthening, similar to that reported previously by Chen et al. [31] in young adult rats. However, in contrast to the latter study, we detected the marked regional differences in transverse myocyte areas, primarily in the noninfarcted free wall myocardium. One potential explanation for such discrepancy might be the fact that Chen and colleagues [31] analyzed cardiac myocytes isolated from both the free wall and the septum together, while in our study, we measured the myocyte cross-sectional areas separately in three LV regions (free wall epi- and endomyocardium; and septal endomyocardium). Besides, our data documenting post-MI compensatory growth of cardiac myocytes in the LV free wall are consistent with numerous previous studies [5,12,13,38,39]. However, in addition to these findings, our data revealed a significant interaction between the effects of sex and the experimental model on myocyte cross-sectional area in the epimyocardial region. Specifically, we established the fact that post-MI female rats, in contrast to males, had no reactive enlargement of cardiac myocytes in the epimyocardial region. Such distinction in myocyte hypertrophic response suggests the existence of a sex-specific difference in regional epicardial wall stress [40,41] between the sexes. Taking into consideration the fact that regionally elevated wall stress can cause pronounced focal degradation of the extracellular matrix [40], it feasible to speculate that such areas of post-MI male hearts may be more prone to side-by-side slippage of myocyte bundles [42,43], increased apoptotic myocyte death [44] and, hence, exaggerated concentric hypertrophy of surviving cardiac myocytes [45].

### Does sex/gender influence a scale of coronary microvessels (capillary and arterioles) adaptation during post-MI remodeling?

Over the past several decades, a large body of accumulating evidence has established the fact that the coronary vasculature undergoes structural adaptations, including angiogenesis and arteriogenesis, in response to postinfarction LV remodeling [46]. However, most of the published experimental studies used either male [39,47-49] or, rarely, female [50] animals only. Most surprising is the fact that, with the exception of our recent studies on middle-aged rats [6,21,22,26], the majority of earlier observations involving murine myocardium have been done on young or young adult mice or rats, which are known to have a more elaborated and highly adaptable coronary vascular network than the hearts of older animals [51-53]. Therefore, our current study is the first to address a sex-specific pattern of coronary microvessel adaptations in the left ventricle of post-MI middle-aged rats.

Consistent with the previous reports on younger animals [39,47,49,54], we found that post-MI hearts of middle-aged rats demonstrated a noticeable reduction in numerical density of coronary capillaries mainly in the regions of the noninfarcted free wall in which the remaining cardiac myocytes underwent substantial concentric enlargement, i.e., in the epi- and endomyocardium of males and endomyocardium of female rats. However, in contrast to males, post-MI female rats had a smaller reduction in capillary density in free wall endomyocardium, despite the fact that both sexes had the noticeable concentric growth of cardiac myocytes in this region. This finding indicates a better regional adaptive expansion of capillaries in the subendocardium of female compared to male rats. Such observation was associated with a significant interaction between the effects of sex and the experimental model on the capillary-to-cardiac myocyte ratio in free wall endomyocardium.

In addition, we found that in both sexes the capillary-to-myocyte ratio increased dramatically only in the areas of free wall associated with enlargement of myocyte cross-sectional area greater than 50% of shame values. Considering the data reported previously on young adult male rats [39], it is feasible to suggest that such a significant increase in the capillary-to-myocyte ratio in these hypertrophic areas of post-MI male and female middle-aged rats, indicates augmented regional compensatory angiogenesis, which could be caused by a variety of factors, including increased wall stress and myocardial stretch, or impaired myocyte oxygenation [10]. Though, the effect of more prominent side-by-side myocyte slippage, which might substantially modify myocyte numerical density in these regions, cannot be ruled out either [38,43].

While the capillary network is important for oxygen delivery to cardiac myocytes, the coronary arteriolar bed is critical for a distribution of blood between capillary domains. Post-MI hypertrophy of the LV myocardium requires structural modifications of the arteriolar tree in order to provide an adequate blood supply to the overloaded myocytes within noninfarcted LV regions [8,9,48]. Unfortunately, nearly all previous experimental studies that addressed arterioles in post-MI myocardium were done exclusively on male animals, including our own earlier studies on middle-aged rats [6,21,22,26]. However, according to our current findings and previous observations on mice of both sexes [55], female hearts have a significantly smaller arteriolar bed than do males. The evident sex-specific differences in the size of the arteriolar bed seen in our study are consistent with the data published by others, regardless of the fact that most of these studies have had the extent of the coronary arteriolar bed reported either for male [56,57] or female [50,58] animals only. The existence of sex/gender related differences in the size of coronary arterial beds has also been confirmed in human studies [59], revealing that women have smaller coronary artery size than men even after adjusting for either body size [60] or left ventricular mass [61].

Despite the fact that arteriolar length and volume densities were significantly greater in male than in female middle-aged rats as indicated by sham values, both sexes demonstrated a relatively similar degree of adaptive arteriolar growth in the LV free wall during post-MI remodeling. However, we found a significant interaction between the effects of sex and the experimental model on the arteriolar diameter and the frequency distribution of arterioles in ‘LV free wall. Specifically, we established the fact that in females, the growth was limited to small diameter vessels only. It is important to note that previous studies done separately on male and female animals have also confirmed the fact that while in post-MI male rats the arterioles grew throughout the entire vascular tree of the remaining LV free wall myocardium [26], in females, the only small size arterioles showed a tendency to expansion [50]. In addition, we uncovered a significant interaction between the effects of sex and the experimental model on arteriolar density in the septum. This finding was associated with the lack of the noticeable compensatory growth of the arterioles in the septum of post-MI female rats as compared to male counterparts. The exact nature of these regional sex-related differences in the responses of male and female coronary arteriolar trees to post-MI remodeling remains to be clarified. We can only speculate that such discrepancy in the pattern of arteriolar bed reorganization within the septum as opposed to the LV free wall of the female post-MI heart, in comparison to males, might be caused by a variety of factors, including the regional difference in wall stress [41] or temporary composition of growth-stimulating milieu [6].

### Study limitations

The current study examined the sex-related differences in hypertrophic response of cardiac myocytes and adaptive expansion of coronary microvessels only at one time point during MI-induced LV remodeling. Therefore, it is difficult to corroborate whether continuing ventricular remodeling would further modify a scale of adaptive responses. Furthermore, because only rats with a large transmural MI were used, the effect of the moderate or small size infarcts on a degree of adaptive processes remained unknown. In addition, it is important to emphasize that this experimental study on middle-aged rats was not intended to investigate the role of sex hormones during a post-MI remodeling process; instead, we attempted to evaluate the responses of different structural components in the remodeled male and female left ventricle under the natural circumstances that might occur in middle-aged humans.

## Conclusions

Taken together our data provided, the first evidence documenting the existence of sex-specific differences in regional adaptation of cardiac myocytes and coronary microvessels in middle-aged rats following 4 weeks of post-MI remodeling. We suggest that such regional peculiarities in structural adaptations within the left ventricle may to some extent influence the sex/gender-related differences in post-MI progression to heart failure.

## Competing interests

The authors declare that they have no competing interests.

## Authors’ contributions

EID designed the study, performed microscopy and digital image collection, evaluated the data, and drafted the manuscript; LPC conducted all experiments and carried out the tissue collection; KO performed morphological assays, including immunostaining and quantitative image analysis; RJT provided financial support to the project, participated in discussion of the obtained findings, and critically reviewed the manuscript. All authors read and approved the final manuscript.
